# Vaccines designed to reduce antimicrobial resistance

**DOI:** 10.2471/BLT.24.020624

**Published:** 2024-06-01

## Abstract

Attention is beginning to focus on the need for vaccines to tackle antimicrobial resistant pathogens. Gary Humphreys reports.

Until fairly recently, debates about how to tackle the looming threat of antibiotic resistance have tended to focus on how to come up with new antibiotics.

Martin Friede, leader of the vaccine research unit at Word Health Organization (WHO) headquarters, would like to broaden the discussion.

“The main mechanisms through which vaccines reduce antibiotic resistance is by preventing infections, both drug-sensitive and drug-resistant,” he says, “but by preventing bacterial infections, vaccines also reduce the need for antibiotic treatments and, consequently, the opportunity for bacteria to develop resistance to antibiotics drugs.”

According to Friede, widespread use of the pneumococcal conjugate vaccine has already significantly reduced the number of antibiotic prescriptions for respiratory infections.

Vaccines that prevent viral infections, such as influenza, also have an indirect impact on antibiotic use because such infections are often treated with antibiotics (creating opportunities for the development of antimicrobial resistance) and/or lead to secondary bacterial infections, which may require treatment with antibiotics.

Finally, while resistance to vaccines or vaccine ‘escape’ does occur, it has tended to develop much slower than antimicrobial resistance.

The relative neglect of vaccines in discussion of antibiotic resistance is reflected in the relatively weak investment in vaccines targeting drug-resistant pathogens.

This was borne out by the 2021 WHO report on vaccine candidates in preclinical and clinical development in the context of antimicrobial resistance (AMR). “The report identified 61 vaccine candidates with relevance for drug-resistant pathogens,” says Friede, “but of the top six bacterial pathogens responsible for deaths due to AMR, only one – pneumococcal disease – had a vaccine.”

Given the strength of the argument for vaccine use in response to antimicrobial resistance, why isn’t there more of a focus on vaccine innovation? One answer is market failure – a term often applied when discussing the challenges faced by developers of antibiotics, but also applicable to vaccines.

Haileyesus Getahun, until recently Director of WHO’s Antimicrobial Resistance Global Coordination Department explains: “Generally speaking, vaccines for use in low- and middle-income countries are not a commercially attractive proposition. They take a long time to develop, are challenging to manufacture and are subject to stringent regulatory controls.”

Moreover, because vaccines are primarily purchased by governments and nongovernmental organizations who are sensitive to price, they tend to offer razor-thin margins, while demand for them shrinks as more of the population is immunized. Demand for vaccines can also fluctuate, spiking during outbreaks but otherwise remaining low, complicating inventory and production management.

“Vaccines reduce antibiotic resistance.”

Martin Friede

As Getahun is quick to point out, where robust markets exist, which tends be in high-income countries, some vaccines have yielded significant returns and pharmaceutical companies have pursued those margins – notable examples including vaccines against the human papillomavirus, influenza viruses, and severe acute respiratory syndrome coronavirus 2 (SARS‑CoV‑2).

While the commercial argument for getting into the vaccine business may be hard to make, the public health argument could not be stronger, as was recently reaffirmed in a WHO-led study published ahead of the 50th anniversary of the Expanded Programme on Immunization which revealed that global immunization efforts have saved an estimated 154 million lives over the past 50 years.

Whether comparable results will be achieved in the future will depend, in part, on making effective use of the vaccines already available, but it will also depend on whether the strength of the public health argument for more vaccine research, including research into drug-resistant pathogens, is allowed to shape the global research and development agenda. 

Encouragingly, there are indications that this may already be happening. These include a sharpening focus on the role vaccines can play, exemplified by the Wellcome Trust study – *Vaccines to tackle drug-resistant infections: an evaluation of R&D opportunities* – published in 2018, which used the WHO priority pathogen list as a starting point for the assessment.

More recently, a meeting of the Product Development for Vaccines Advisory Committee suggested that national governments are also keen to prioritize efforts targeting drug-resistant infections.

Established by WHO to provide guidance on priority areas for vaccine research and development, the committee last met in December 2023 to identify pathogen priorities for new vaccine development based on extensive consultation with national governments in the six WHO regions.

The meeting resulted in the drawing-up of a global priority pathogen list comprising diverse pathogen classes. Of the 17 chosen, four bacteria had demonstrated multi-drug resistance, namely: extra-intestinal pathogenic *Escherichia coli*; *Klebsiella pneumoniae; Staphylococcus aureus; and Mycobacterium tuberculosis*.

The committee also took note of the significant changes brought about by the coronavirus disease 2019 (COVID-19) pandemic. These included a sharpened focus on the potential of vaccines and new vaccine technologies to counter disease outbreaks, such as the messenger ribonucleic acid (mRNA) platforms that were instrumental in the development of vaccines against COVID-19, and the vital importance of committing resources to vaccine research and development.

“There was a recognition that systematic investment in the entire vaccine R&D ecosystem is needed to effectively and equitably respond, not only to the next pandemic, but to other priority diseases for which we currently do not have vaccines or where vaccine product innovation is needed,” says Getahun.

“Systematic investment in the entire vaccine R&D ecosystem is needed.”

Haileyesus Getahun

Erin Duffy, Chief of Research & Development at CARB-X (Combating antibiotic-resistant bacteria), a global non-profit partnership dedicated to accelerating antibacterial research, also reports a shift in attitudes.

“Since the pandemic there has been a heightened recognition of the indispensable role prevention plays in public health,” she says, underlining the importance of proactive measures to curb the spread of infectious diseases, “including those displaying antimicrobial resistance.”

According to Duffy, CARB-X included a specific call for vaccines and other biotherapeutics proposals in its 2019 funding round and is backing a vaccine – GlyProVac – as one of the investments the partnership is making this year for vaccines to prevent neonatal sepsis.

“Neonatal sepsis is a life-threatening response to bloodstream infections that occurs in newborns fewer than 28 days old. Due to their immature immune systems, newborns are particularly susceptible to infections,” Duffy explains.

GlyProVac targets *Escherichia coli*, which causes a large proportion of neonatal sepsis. Because neonates are too young to be vaccinated, the vaccine will be given to mothers who will then pass their acquired immunity to the unborn child.

Notwithstanding such efforts – and significant research and development on human immunodeficiency virus, tuberculosis and malaria, all of which also develop resistance to available treatments – relatively little effort is going into vaccines against so-called superbugs.

It is a state of affairs that is particularly frustrating for innovators who feel they have game-changing products sitting in their laboratories.

Bruno Santos is one of them. Chief executive officer of a Portuguese biotech start-up based in Cantanhede, Portugal, he and his colleagues have two products in development – one of which has, in his words, “the potential to protect recipients against five multidrug-resistant bacteria.” Three of those bacteria are on WHO's priority pathogens list: *Escherichia coli; Staphylococcus aureus; *and* Klebsiella pneumoniae.*

Each of these bacteria actively suppresses the human immune system by excreting a protein which engages with the cells primarily responsible for producing antibodies. For the past 11 years, the immunologists in the start-up have been studying this process and, according to the results of successful preclinical trials, may have found a way to block it.

The company is keen to start human trials but lacks the funding needed to proceed. “We have talked to several big players in the pharmaceutical sector and there is a lot of interest, but securing initial investments remains a challenge,” Santos says.

Santos would like to see governments, including the Portuguese government, increase their investment in the kind of research his company is doing.

Countries also have the option of offering tax incentives to reduce the cost burden and encourage investment, while advanced market commitments can be used to guarantee sales. In this regard, it is worth pointing out that the pneumococcal conjugate vaccine advanced market commitment, launched by Gavi, the Vaccine Alliance, in 2009 with the support of the donor governments, vaccine manufacturers and other stakeholders, played a vital part in ensuring access to pneumococcal conjugate vaccines in developing countries. The Gavi COVAX AMC launched in June 2020, played a similar role in ensuring the supply of COVID-19 vaccines free of charge to 87 low- and lower-middle-income economies.

Santos also argues for accelerated trial schedules, as were used in the development of some COVID-19 vaccines, and for regulatory bodies to expedite review and approval processes. 

His company is planning to go through European Medicines Agency and United States of America Food and Drug Administration approval once his vaccine has made it through clinical trials.

Whether or not the vaccine makes it through those trials is, of course, an open question – but it is a question that will not even be asked if the company and others like it cannot raise the money to run them.

**Figure:**
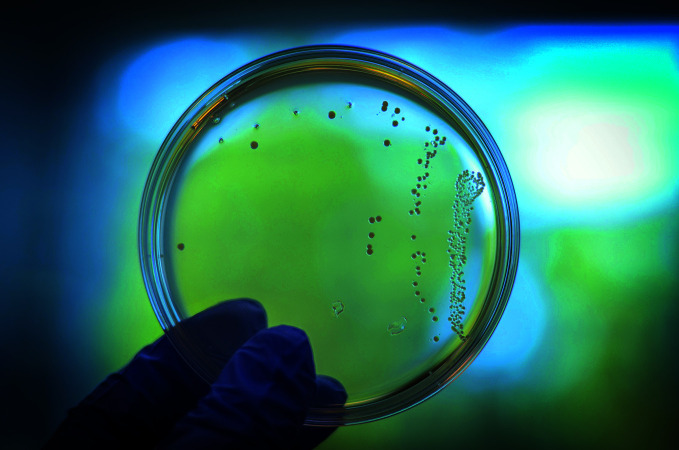
Bacteria reacting to a candidate vaccine in a petri dish

Immunethep

**Figure:**
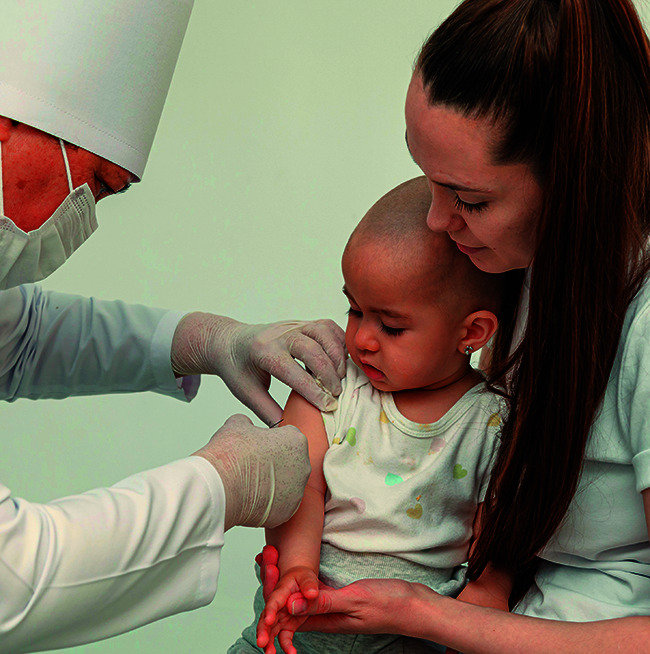
A health worker vaccinates an infant in Ashgabat, Turkmenistan

WHO/Pavel Belyayev

